# In memory of Jay A. Siegel, PhD, 1946–2017

**DOI:** 10.1080/20961790.2018.1442644

**Published:** 2018-03-26

**Authors:** Douglas H. Ubelaker

**Affiliations:** National Museum of Natural History, Smithsonian Institution, Washington, DC, United StatesUBELAKED@si.edu

On September 25 2017, Jay A. Siegel, PhD died at his home in Mt. Arlington, New Jersey at the age of 71. At the time of his death due to heart failure, Dr Siegel was highly regarded internationally as a forensic chemist, educator and prominent advocate for quality in the forensic sciences. Jay was born in Washington, DC, USA on April 16 1946. He received a PhD degree in chemistry from The George Washington University in Washington, DC, USA in 1975. Early in his career, Jay developed strong interest in the forensic applications of chemistry. Following employment as a forensic chemist with the Virginia Bureau of Forensic Sciences, Jay launched decades of professional activity as an educator and research scientist with Metropolitan State College in Denver, Colorado, Michigan State University in East Lansing, Michigan and Indiana University-Purdue University in Indianapolis, IN, USA. His university experience included serving as department chair but primarily focused on educating many undergraduate and graduate students who have in their own subsequent careers made many contributions to forensic science. His international recognition included visiting professor roles at universities in Australia and China.

Jay received many prestigious honours and awards during his long career. These include the Paul L. Kirk Award in 1975 from the Criminalistics Section of the American Academy of Forensic Sciences (AAFS). Jay was named Distinguished Fellow by the AAFS in 2009. Also in 2009, he received the Distinguished Alumni Award from The George Washington University and in 2012 the Distinguished Service Award from the Midwest Association of Forensic Scientists. Jay served on the editorial boards of the *Journal of Forensic Sciences* from 1983 to 2008 and the *Forensic Sciences Research* from 2016 to 2017 and as a commissioner for the Forensic Science Education Programs Accreditation Commission (FEPAC) for six years beginning in 2003.

Throughout his career, Jay was a strong advocate for quality and scientific rigour in the practice of forensic science. He was selected to serve on the National Research Council Committee that led to the 2009 publication of the influential “Strengthening Forensic Science in the United States: A Path Forward.” Jay authored books and numerous publications in relevant scientific journals within his interests in forensic chemistry and the forensic sciences in general. He was an editor (with Pekka J. Saukko) for the Encyclopedia of Forensic Sciences published in 2012 by Academic Press.

Over the years, I enjoyed many discussions with Jay regarding issues within the forensic sciences and developments relating to AAFS. He could always be counted upon for strong, thoughtful positions on topics of mutual interest. From 2008 to 2016, I served as the Book Review Editor for the *Journal of Forensic Sciences*. During this time, I recall many conversations with Jay concerning the quality of publications in the forensic sciences, especially relating to his own specialty of forensic chemistry. In my additional role as Chair of the Forensic Science in Focus Book Series for AAFS, I called Jay, challenging him to organize a quality, edited book on forensic chemistry to be published in the series that would address many of the shortcomings we had discussed previously. Jay accepted this challenge and responded with a carefully edited manuscript with contributions from many of his colleagues. The volume “Forensic Chemistry: Fundamentals and Applications” was published by Wiley-Blackwell in 2015.

In my view, Jay Siegel represents an ideal role model for all of us in the forensic sciences. His work demonstrated how academia and forensic practice are not mutually exclusive. Like Jay, we should all strive for the highest quality in forensic science possible and embrace the challenges of constructive criticism and the advances of new research.

